# “Sharp downward, blunt upward”: district maternal death audits’ challenges to formulate evidence-based recommendations in Indonesia - a qualitative study

**DOI:** 10.1186/s12884-021-04212-7

**Published:** 2021-10-27

**Authors:** Ratnasari D. Cahyanti, Widyawati Widyawati, Mohammad Hakimi

**Affiliations:** 1 Obstetrics and Gynecology Department, Faculty of Medicine, Diponegoro University, Semarang, Indonesia; 2 Doctoral Program, Faculty of Medicine, Public Health and Nursing, Universitas Gadjah Mada, Yogyakarta, Indonesia; 3 Pediatric and Maternity Nursing Department, Faculty of Medicine, Public Health and Nursing, Universitas Gadjah Mada, Yogyakarta, Indonesia; 4 Obstetrics and Gynecology Department, Faculty of Medicine, Public Health and Nursing, Universitas Gadjah Mada, Yogyakarta, Indonesia

**Keywords:** Maternal death, Audit and review, Evidence-based recommendation, Indonesia

## Abstract

**Background:**

Indonesia, the largest archipelago globally with a decentralized health system, faces a stagnant high maternal mortality ratio (MMR). The disparity factors among regions and inequities in access have deterred the local assessments in preventing similar maternal deaths. This study explored the challenges of district maternal death audit (MDA) committees to provide evidence-based recommendations for local adaptive practices in reducing maternal mortality.

**Methods:**

A qualitative study was conducted with four focus-group discussions in Central Java, Indonesia, between July and October 2019. Purposive sampling was used to select 7–8 members of each district audit committee. Data were analyzed using the thematic analysis approach. Triangulation was done by member checking, peer debriefing, and reviewing audit documentation.

**Results:**

The district audit committees had significant challenges to develop appropriate recommendations and action plans, involving: 1) non-informative audit tool provides unreliable data for review; 2) unstandardized clinical indicators and the practice of “sharp downward, blunt upward”; 3) unaccountable hospital support and lack of leadership commitment, and 4) blaming culture, minimal training, and insufficient MDA committee’ skills. The district audit committees tended to associated maternal death in lower and higher-level health facilities (hospitals) with mismanagement and unavoidable cause, respectively. These unfavorable cultures discourage transparency and prevent continuing improvement, leading to failure in addressing maternal death’s local avoidable factors.

**Conclusion:**

A productive MDA is required to provide an evidence-based recommendation. A strong partnership between the key hospital decision-makers and district health officers is needed for quality evidence-based policymaking and adaptive practice to prevent maternal death.

## Background

The World Health Organization (WHO) launched the maternal death surveillance and response (MDSR) program in 2013 for strengthening the follow-up action of maternal death reviews (MDR) to prevent future preventable maternal deaths [1, 2]. This program uses a continuous-action cycle of identification, quantification, mandatory notification, and review of causes and avoidable factors of maternal death followed by recommended action and monitoring [3, 4]. Through this cyclic process, MDSR mirrors the steps of a typical audit or quality improvement cycle. As an integral part of MDSR, MDR is of strategic importance for evidence-based policymaking in local contexts to reduce preventable maternal death [4–6].

In Indonesia, the MDR is commonly called a maternal death audit (MDA) has been implemented since 1994. A national guideline adopting confidential enquiry into maternal death (CEMD) was implemented in 2010 and revised in 2015. This guideline sets outlines for reporting maternal and perinatal deaths, implementation of death review, and action recommendations as a socio-accountability of the local government. In 2016, the Ministry of Health of the Republic of Indonesia initiated more proactive maternal death surveillance and launched a revised guideline by improving the identification and reporting of maternal death [7, 8]. Despite the implementation of MDA, the MMR in Indonesia remains higher than those of neighboring countries in the ASEAN region [9]. The Inter-Census Population Survey (SUPAS) announced that the number of MMR in Indonesia in 2015 was 305 deaths per 100,000 live births, significantly higher than the Millenium Development Goals (MDGs) target of 102 per 100,000 live births [10]. However, the Maternal Mortality Estimation Inter-Agency Group (MMEIG) released a trend that estimated Indonesian MMR at 177/100,000 live births in 2017 [11]. Being a lower middle-income country, with < 40% countries have proper civil registration (CR) records to determine accurate and synchronous data, calculating MMR in Indonesia remains a challenge. Nonetheless, the number of maternal deaths in Indonesia is still high, and 90% of them are preventable maternal deaths [12–14].

Accelerated efforts to reduce maternal mortality include delivering antenatal service, training, and expanding health facilities in basic and comprehensive Emergency Obstetric Care (EmOC) services, strengthening public health systems, promoting community health insurance, and mobilizing community awareness. However, there have been challenges to ensure timely treatment and adequate care for obstetric complications at health facilities associated with the disparity factors among regions, such as geography, ethnicity, and cultural norms [13, 15–17]. In Indonesia, with decentralized health systems, the full mandate of authority to provincial and district levels proposes the effort to improve maternal health services in each district [18]. The system offers solutions for those challenges, and implementation of maternal audits and reviews at the district level are crucial to identify local challenges by conducting local assessments instead of national reviews to support faster policy changes [19, 20].

In an effort to improve the quality of MDA implementation at the district level, the Republic Indonesia Ministry of Health has facilitated MDA implementation guidelines at the district level with periodic revisions. National MDA guideline explains the informative task of the MDA committee and the steps for implementing the maternal death audit and review. To support the decentralized health system program, the local government, as stated in the national MDA guidelines, involves all the district government stakeholders, including the Major/Regent as the steering committee of MDA. Their primary responsibility is to provide a legal framework and policy for those involved in the management (DHO), reviewer team, and health service community. The legal framework includes a decree of the district MDA committee and MOU between district health facilities and government stating the mutual relationship between district audit committees and health facilities, requiring the health facilities to provide notification and submit the maternal audit form to obtain the audit feedback from district MDA committee. The national guideline also advises reviewers conducting maternal mortality case studies to use scientific evidence to formulate recommendations. It is hoped that the implementation of maternal death audit and review at the district level will assist in developing local policies with the translation of national health guidelines and policy into specific interventions according to the problems in the region [7].

This present study is an effort of local assessment by exploring the district maternal death audit committees’ views and experiences in formulating evidence-based recommendations and action plans. Recently, no studies have rigorously highlighted the key challenges to develop evidence-based recommendations for local adaptive practices to reduce preventable maternal mortality in the national decentralized health system settings.

## Materials and methods

### Study design

This was a qualitative study which involved district maternal death audit committees in focus group discussions (FGDs).

### Study setting

The study was conducted in four districts (Brebes, Pekalongan, Kendal, and Grobogan) with high numbers of maternal deaths in Central Java, Indonesia. The Central Java province has 35 districts; each district has different numbers of maternal mortality cases. Central Java DHO encourages each district to report the absolute number of maternal deaths rather than the calculated MMR. Indonesian Ministry of Health urges devising intervention to reduce maternal mortality; based on MMR and the number of maternal mortalities in high population areas. Central Java constitutes around 15% of Indonesia’s population and is one of the provinces with a high number of maternal mortality cases [21]. In 2015, the final year of MDGs, this province contributed 12.87% (619 cases) to the total number of national maternal death (4809 cases) [22, 23]. To reduce maternal death, the public health office of Central Java has conducted an annual program to provide supervision and financial assistance for district MDA with external reviewers from the Indonesian Society of Obstetrics and Gynecology (POGI) as national obstetrician-gynecologists experts.

### Sampling method

A maximum variation sampling technique was applied to select districts in order to include a wide range of variation in the geographical region, regional income, number of populations, and health facility ratio.

We mainly selected the members of district audit committees, appointed through an MDA decree signed by a respective Major of a city or a Regent of a regency, responsible for conducting and organizing regular MDA. The MDA team consists of a management team (DHO) and internal reviewers. Internal reviewers are experts representing professional organizations in the region and are often actively involved in patient management in hospitals in the area. In addition to conducting a maternal death review, internal reviewers also formulate recommendations and action plans addressed to the DHO management team. DHO management team then implements the plans according to the targets of each of these recommendations.

Two areas in DHO, the health service department and the public health department, combined their efforts to improve the quality of maternal health services in the decentralized health system in the region. These two play an essential role in organizing MDA at the district level, called the MDA management team. The head of the public health department serves as an MDA coordinator to manage the district MDA and, with their team, coordinates the data collection, review, supervision, and monitoring. Meanwhile, the head of the health service and the staff support the duties of the health office as the primary coordinator of MDA, notably in implementing the recommendations in improving the quality of services in health facilities.

This study recruited 29 informants who participated in four FGDs. Each FGD participant involved the same composition included:

- Internal reviewers consist of two obstetrician and gynecologists and one midwife.
- A management team from the District Health Office (DHO) consists of the health service and public health head departments with their respective staff. The public health department’s staff, which acts as the MDA secretariat, manages data collection. In addition, the health service department’s staff supervises health services quality improvements in public health centers and hospitals.

### Data collection

The topics of the FGDs were based on the existing literature about related disabling factors of MDA for evidence-based policy making [20, 24, 25]. The FGD topics were as follows:

- How do you review the causes of maternal death?
- How do you develop recommendations for prevention programs in your district?
- How do you implement the recommendations of MDA in your district?

The FGD guidelines were tested by a researcher (RC) and a research assistant in the pilot study. The results of the pilot FGD were discussed with the other authors for improvements.

The four FGDs were conducted from July to October 2019. The FGDs lasted for 1–2 h. To ensure their convenience, the FGDs were conducted in a small room meeting at a local café or restaurant based on the agreement between the researchers and informants. All the FGDs in Bahasa Indonesia were led by the first researcher and a research assistant. An observer used field notes to record participants’ non-verbal aspects during the FGDs.

### Positionality, reflexivity, and triangulation

Maternal death is a sensitive issue, and the research topic has the potential to cause social consequences. The first researcher has had an engagement with the MDA team in the district. The first researcher is one of the external reviewers of POGI, participated in several activities to reduce maternal mortality with DHO and Central Java Provincial Health Office, and is an EmOC trainer in Central Java. Previous encounters built trust within the MDA district committee and thus identified key informants recruited as focus group data collection participants. Member checking was performed to reduce bias of description and interpretation. The findings from the discussions were reevaluated with the other researchers to review and understand the data. In addition, peer debriefing was carried out between the researchers and external reviewers, representatives of POGI in Central Java with relevant expertise. To assess the progress of the maternal audit implementation in the district, the researchers also reviewed the audit documentation of maternal mortality cases in 2018 and 2019 in each district.

### Data analysis

The FGDs were recorded and transcribed verbatim. The first researcher led and conducted the thematic data analysis to investigate both the manifest and the underlying meanings of the texts focusing on the challenges in developing recommendations and action plans of the MDA. During the coding process, we used key concepts of organizational change to translate CEMD into the implementation of recommendations and persistently identified new information from the data [24]. In the first stage of analysis, the meaning units of the transcripts were identified to generate manifest meanings. The manifested meaning units were then coded. The codes were grouped into subcategories or categories based on their meanings (see Table 1). The transcripts of FGDs were analyzed using NVivo 12 software (QSR International, Melbourne).

**Table 1:** An example of the coding process, from meaning units to subcategories

Topics	Meaning units	Condensed meaning unit	Codes	Sub-categories
How do you develop recommendatios for prevention programs at your district?	“Sometimes the data obtained using the instruments were not accurate due to data falsification leading to inaccurate recommendations.”	Distrust to the maternal death data by MDA committee due to accuracy concerns (falsification) leading to an inaccurate recommendation	-Accuracy of maternal death data -Data falsification -Distrust data -Inaccurate recommendation	Inaccuracy
How do you implement the recommendations of MDA at your district?	“We involved a team of reviewers to develop recommendation to implement, the challenges is that the higher-level of health facilities [the target of recommendation] sometimes do not adhere to all of the recommendation”	The collaboration between officials of DHO and reviewer in developing recommendation, challenges in the implementation of recommendation related to adherence of higher-level of health facilities	- Collaboration - Challenges - Adherence of higher-level health facilities - Recommendation -Adherence concerns to MDA recommendation	Adherence

### Ethical considerations

Ethical approval No. 102/EC/KEPK/FK UNDIP/2019 for this study was obtained from the Research Ethics Committee, Faculty of Medicine, Diponegoro University, Semarang, Central Java. Permission and approval to conduct the study were received from the Government of Central Java and Brebes, Pekalongan, Kendal, and Grobogan District Health Offices, Central Java. Prior to the FGDs, the researchers explained the aim of the study and topics under discussion, confidentiality of information, and informants’ right to withdraw at any time. Written informed consent for audio-recording and using excerpts in publication and reports was obtained prior to the FGDs from all participants. The informants were anonymous during the analysis and presentation of results.

## Results

Of 29 informants from four FGDs, most participants (69%) had 1–3 years of experience participating as a district audit committee member (Table 2). Most informants were 40–50 years old (55.2%) and had a Master’s degree in Public Health (44.8%). We identified the main challenges to formulate evidence-based recommendations and action plans for the district MDA, including: 1) non-informative audit tool provides unreliable data for review; 2) unstandardized clinical indicators and the practice of “sharp downward, blunt upward”; 3) unaccountable hospital support and lack of leadership commitment, and 4) blaming culture, minimal training, and insufficient MDA committee’ skills. Table 3 shows the data analytic framework of the study.

**Table 2:** Characteristics of participants

Characteristcs	***N*** = 29	%
**Age**		
30–40 years	3	10.3
40–50 years	16	55.2
50–60 years	10	34.5
**Gender**		
Male	12	41.4
Female	17	58.6
**Education**		
Obstetrician and Gynecologist	7	24.1
Medical Doctor (MD)	4	13.8
Master’s degree in Public Health	13	44.8
Diploma of midwifery	5	17.2
**District audit committee members**		
Grobogan	7	24.1
Brebes	8	27.6
Kendal	7	24.1
Pekalongan	7	24.1
**Experience (audit committee)**		
1–3 years	20	69.0
> 3 years	9	31.0
**Audit committee**		
Management team	15	51.7
Internal reviewer	14	48.3

**Table 3:** Data analytic framework

Theme	Categories	Sub-categories	Codes
Non-informative audit tool provides unreliable data for review	Inadequate instrument	Irrelevant information	Irrelevant information from maternal death instruments
		Inadequate forms	Inadequate and/or reliable maternal audit forms
		Incomplete instruments	Instruments cannot provide all the necessary information surrounding maternal death
	Supporting informative data	The need for supporting data	The reviewers urged supporting data
		Collecting additional document	Collecting data staff providing additional documents in maternal care
	Inaccurate information	Inaccuracy	Out of sync information in referral case
			Reviewers distrust the data provided by the hospital
			Unreliable data
			Accuracy of maternal death data
			Data falsification
Unstandardized clinical indicators and the practice of “sharp downward, blunt upward”	The ignorance of the reviewer to use clinical standards to identify the gap	Clinical experience	Review based on clinical experience only
		“Medicine is an art “perspective	Review based on the belief that medicine is an art
		Personal perception	Review based on the personal perceptions
		Tendency to associate the problem in the lower-level health facility	Reluctant to review the case involved senior colleagues
			“Sharp downward, blunt upward”
	Personal initiative to use clinical standards for an objective review	An objective review	The external reviewers are more objective
		A personal initiative by an external reviewer to use clinical guideline	An initiative of the external reviewer to use the national clinical guideline to identify the problem
Unaccountable hospital support and lack of leadership commitment	Lack of commitment to the implementation of the role of audit	Inadequate support of the management team to the role of audit	Failure to comply with the terms of agreement of MDA
			DHO needs an advocacy process involved the external review
		Failure to comply with proactivity in providing information	Failure to provide information of maternal death
			Challenging communication to obtain data from hospital
	Difficulty of DHO to implement the recommendation to the hospital	Lack of recognizance to DHO authority	Hospital decision-makers disrespect to the DHO team
			Poor awareness of DHO of their authority over the hospitals
		Lack of commitment to attend and understanding the audit feedback	Poor attendance of hospital decision-makers to audit meeting
			Absence of adapted practice based on recommendation
		Adherence	Collaboration to implement recommendation
			Challenges in the implementation
			Adherence of higher-level health facilities
			Recommendation to hospital
Blaming culture, minimal training, and insufficient MDA committee’ skills	Failure to internalize the principles of audit		*‘*Blaming culture’, leading to the reduction of a set of review processes into merely a ‘disciplinary process’
			Punitive actions by reviewers in terms of revealing personal and institutional information to the public
	Lack of knowledge to program an MDA		Insufficient training of audit committee Incompatible education background
			Lack of training
			Frequent staff rotation
	Failed to translate recommendation into policy		Lack of specificity of recommendation Absence of cross-sectoral partnership between stakeholders Poor budgeting allocation

### Non-informative audit tool provides unreliable data for review

To accurately identify the cause of maternal death and its contributing factors, the district MDA committees must obtain relevant information from maternal audit forms. However, these forms are likely inadequate and unreliable. In terms of inadequate forms, one of the reviewers stated that relying on the available instrument cannot provide all the necessary information surrounding maternal deaths. He explained that the item of antenatal care in the instrument merely provides information on the number of antenatal visits without clarifying in detail about the care provided.

*“Honestly, when conducting a review, I cannot get the big picture of the cases using the instruments [maternal audit forms]. We can get the big picture from the chronology of maternal death provided by the caregivers, patient relatives, instead. Sometimes, we contacted the midwife responsible for ANC because MDA often easily concluded with poor ANC. However, the instrument [maternal audit forms] only included the question on the number of ANC [visits].”*

(An internal reviewer, 49 years old)

To solve these problems, providing supporting data for evidence-based policymaking was urged by the reviewers. They proposed the collecting data staff to provide them with additional documents including the records of the antenatal, intrapartum, and postpartum care provided.

*“ … we [are assigned to] add the complete information surrounding maternal death including antenatal care, disease history, etc., in a different file compilation, both in soft and hard copy.”*

(A data collector (management team), 42 years old)

The district MDA committees noted that the other challenges on understanding completed maternal audit forms in referral cases involve several health facilities providing out-of-sync information surrounding maternal death. Some reviewers also believed that data provided by higher-level facilities tended to be unreliable. They also expressed their preference for the maternal death data provided by the staff of lower health facilities compared to those of higher levels.

*"From how it's usually approached, the hospital is often blamed. So, in the end, the data that was sent was not as is. The internal audit isn’t being conducted, as a result, the information created on the form is often raw data written by the lower staff. All I did was sign it, and it was sent without being discussed internally.”*

(An internal reviewer, 35 years old)

### Unstandardized clinical indicators and the practice of “sharp downward, blunt upward”

There was a disagreement among the reviewers on how to conduct an MDA review due to no explicit clinical standard available. The majority of the internal reviewers perceived that their own clinical experiences and personal perceptions were the main consensuses to identify gaps and highlight deficiencies of maternity care. For example, some reviewers perceived that a clinical standard or evidence-based guideline is of strategic importance in identifying major significant gaps between the care that was given and the care that should have been given. However, some considered medication as an art:

*I (Interviewer): “How did you analyze a case of maternal death?”*

*Respondent (R)6: “So far, we did it based on our knowledge.”*

*R1: “Let me give you an example, in the administration of misoprostol, in the same case, different patients were given different doses, sometimes two tablets, one tablet, and half a tablet.”*

*R3: “Oh, for reviewing the case. I guess..”*

*R6: “Yes, to determine if there was an overdose or not.”*

*R3: “An explicit standard should have been available.”*

*R6: “For developing a recommendation.”*

*R3: “When I did my practice in district A, all the Obstetricians did not dare to give misoprostol, they preferred oxytocin for induction of labor.”*

*R5: “Yes, now we give oxytocin. The misoprostol should not have been given to primipara. Yes, even though it is an art too.”*

*R6: “Art is difficult to audit.”*

(FGD1, MDA committee)

Some internal reviewers expressed doubts about their findings related to the primary cause of death and contributing events in higher-level facilities. They admitted that the seniority of their colleagues in higher-level facilities prevented them from giving their objective review. They recalled a common situation when the internal reviewers felt it was inconvenient to reveal the practice deficiencies done by their senior colleagues responsible for the patient’s death.

*“Well, the problem is, sometimes there are hospitals with senior obstetrician and gynecologists. He was the main barrier, when he said something, nobody dared to argue, it’s difficult. It would have been much easier to identify the truth if an explicit standard had been available.”*

(An internal reviewer, 51 years old)

In such situations, the internal reviewers would associate the cause of maternal death under review with the mismanagement of the lower level of health facilities involving midwives and staff of the primary health centers.

*“The reviewer tends to blame the health providers at the lower facilities.*”

(A management team member, 50 years old)

The tendency was described by one informant using an Indonesian proverb (traditional saying) as “… this *[tendency]* is like “*tajam ke bawah, tumpul ke atas,*” translated into English as “sharp downward, blunt upward.” Furthermore, the informant also gave an example of a case in which the mismanagement of maternal care was attributed to a midwife even though no maternal risk was found in the midwife’s initial assessment.

*“When there was a maternal death, the review resulted in the failures of the midwife in providing care. I got information from my friends, there were cases in which the patient was managed according to the procedure, this patient did not have any risk, but she insisted on being referred to the hospital, and the patient died there, but the review stated that there was a midwife failure.”*

(A management team member, 50 years old)

One member of the management team expressed her opinion on the objectivity of external and internal reviewers in conducting MDR. External reviewers are perceived to be more objective than internal reviewers because they referred to the national clinical guidelines to provide evidence-based recommendations, for example, the district program of calcium supplementation in high-risk pregnant women to prevent preeclampsia.

*“They, the external reviewers from the education center, gave a recommendation based on the national clinical guidelines. It includes the recommendations for calcium administration to prevent preeclampsia. This recommendation is not from us [the internal reviewers].”*

(A management team member, 44 years old)

In this context, the use of the national clinical guideline in MDR was based on personal initiative. The national MDR guideline does not explicitly require the reviewer to refer to a specific clinical guideline.

### Unaccountable hospital support and lack of leadership commitment

According to the Indonesian MDA guideline, health facilities and the district health office (DHO) should have a reciprocal relationship. The former is responsible for providing information surrounding maternal death, and the latter is accountable for giving feedback. In the implementation, to ensure the strong commitment to achieving successful MDA, the DHO and hospitals entered into a Memorandum of Understanding (MOU). However, there was a failure to comply with the terms of the MOU agreements indicating poor support. One management team member mentioned the inability of the hospital’s decision-makers to comply with proactivity in providing information surrounding maternal death. She shared her profound efforts to obtain the required data, including physically going to the hospital and conducting the correspondence herself. It was even more challenging to obtain data of maternal death occurring in the hospital outside of the patient’s residency area (stated in her family identity card). The absence of mutual communication leads to the failure to comply with the regular audit schedule.

*“… There was a maternal death in the town [outside of her residency stated in ID card] in a private hospital. Since January, we have visited and sent letters to the hospital several times. [after seven months] we haven’t received maternal death audit forms. I do not know what to do. We have entered into MOU, I cannot think of any other ways to communicate with them [hospitals].*

(A management team member, 45 years old)

Another poor support from key health decision-makers was indicated by their lack of commitment to implement the recommendations. Early commitment can be shown by their presence, while most of the time, they were absent or had a representative from the lower range staff to attend the meeting. Thus, there were no two-way discussions between reviewers and the reviewee for the sake of better outcome implementation. The further commitment is indicated by initiating adapted practice in their work settings. A reviewer putting himself in the role of the hospital under review admitted that despite knowing the problem, the key health decision-makers are reluctant to consider strategies and customize them to ensure the implementation of recommendations in their work settings.

*“At least we know there has been a delay in site A [for eight years]. We actually know what to do in half an hour [response time]. The problems, the providers are reluctant to implement the recommendation. For example, in the district hospital, there are no health personnels on emergency duty in the operating room … .I don’t think that the health personnels are available in the operating room at night.”*

(An internal reviewer, 45 years old)

The policymakers often assume that translating evidence of the causes of maternal deaths is a linear process. This poor support is amplified by the poor awareness of the hierarchy of authority. For example, district health officers were not aware that DHO has a higher authority than the hospitals in the health system organization. This contributes to the non-compliance of the hospital’s key health decision-makers to the recommendations. In fact, some decision-makers were disrespectful toward the DHO team. In addition, the district health officers themselves perceived that external reviewers have more substantial authority to regulate hospitals. Thus, to prevent disrespectful attitudes, the DHO expressed their need for the presence of external reviewers to persuade the hospital to implement the recommendations.

*” … .Sometimes when we report the MDA findings to the hospital, they reject them. I feel like I have no bargaining position, that I have no power. I wish I were with the external reviewers so it can be more objective.”*

(A management team member, 50 years old)

### Blaming culture, minimal training, and insufficient MDA committee’ skills

To achieve the goal of MDA to prevent similar preventable maternal deaths, the concept of CEMD adopted in MDA is intended to avoid the fear of health workers about punitive actions to ensure the complete information needed for formulating recommendations. However, these ‘no name, no shame, no blame’ principles have not been well internalized. A member of the MDA committee revealed there was still a ‘blaming culture’ leading to the reduction of a set of review processes into merely a ‘disciplinary process.’ In addition, one member of the management team mentioned one of the examples of punitive actions by reviewers involved revealing personal (health workers) and institutional (health facilities) identities to the public, disrespecting and violating their right to anonymity.

*“… , I can tell that health facilities under review are concerned with the possibility of publicity. Thus, the data reported to the [District Health] Office is not really [reliable], … when they got a warning, they would not take it, that’s why it is imperative to implement the principles of no name. As we may know, the goal is to offer solutions. However, some health facilities showed anger, they perceived us as a judge blaming a case.”*

(An internal reviewer, 49 years old)

The district MDA committee admitted that the lack of internalization was due to inadequate knowledge to conduct evidence-based policy-making. The cause of lack of expertise varied among different members of the MDA committees. The leading reason for the lack of knowledge for the management team was insufficient training and incompatible education background. After the training on MDA organized by the Central Java Health Office, the members of district MDA committees, including the Head of the Public Health Department, received the decree for MDA committee appointment. Unlike the previous government official, a newly appointed Head of the Public Health Department admitted that she had never attended training on MDA before she coordinated district MDA.

*“The MDA decree was dated in 2016, and I just continued [as coordinator of MDA] and I was appointed [the new Head of Public Health Unit] in late 2017.*”

(The management team, 45 years old)

In one of the FGDs, a Head of the Health Service Department self-reported his incompetency because he had no education background in health service management. The head of the Public Health Service as his partner in the DHO, confirmed his incompetency indicated by failing to serve the function as an official government authorized to manage MDA in the health facilities. She noted that she personally decided to take over the task of the Health Service Department in regulating health service management in the lower-level health facilities but not in higher-level facilities.

*I wish [health service] to play this role shows that the Public Health Department is not willing to take all the responsibility [including implementation recommendation]. I cannot ensure an optimal outcome because we have no authority [to regulate] hospitals.”*

(A management team member, 44 years old)

The main identified causes of lack of knowledge for internal reviewers were insufficient training and/or knowledge related to the MDA national guideline. A ‘junior’ internal reviewer admitted that he had not attended any training on MDA. Since the ‘senior’ reviewer tended to hand over his tasks to the ‘junior’, he wished that he had a transfer of knowledge from his trained ‘senior’. Another internal reviewer also admitted that he had no knowledge related to the MDR guideline. In fact, he had no idea about the publisher and what is covered in the MDR book.

*“On the bottom line, there has been no standard in place. My partner and I haven’t attended any audit workshop. After all this time, we rely on our clinical expertise. I also asked the input from my colleagues. Honestly, [I do not know] the audit rule and the guideline. For example, in our district and neighboring districts, instead of the juniors [the backbone of MDA] few seniors attended the workshop.”*

(An internal reviewer, 49 years old)

Unlike the management team and reviewers, the community health care teams in public health centers had conducted sufficient training. However, an overlapping policy or mismanagement of human resources at the district level indicated by frequent staff rotation leading to lack of knowledge. It implies that by not having received a specific decree of appointment, the new staff replacing the rotated staff had none of the required knowledge and skills for their new job description.

*“… ..we have annual refreshing training, however, this year we don’t have one. The staff of the Public Health Center’s problem is frequent rotation. The head of the district health office keeps rotating the staff, including those who have been appointed as [a collecting data staff] of MDA.”*

(A management team member, 45 years old)

The MDA committee’s lack of knowledge had an impact on the institutionalization of recommendations. The MDA committee had failed to internalize the principle of adherence to the action plans and advocacy strategy. The MDA committees highlighted that the MDA merely concludes with identifying the causes of maternal death. It is difficult, if not impossible, to implement the recommendations due to the lack of specificity.

*“Most of the time, I am not sure what to recommend.”*

(A management team member, 58 years old)

There has been a shift in the main contributing factors of maternal deaths from delay in deciding to seek care and reaching a health facility (demand-side barrier) to delay in receiving quality care (supply-side barrier). A reviewer perceived that the MDA program does not achieve its goal because the head of the district office failed to translate the recommendations into policy-making. Another head of the Public Health Department expressed her concerns over the absence of cross-sectional partnership between stakeholders (public health program and health service program) to implement the recommendations. A reviewer revealed a supply-side barrier due to poor job description sharing leading to failure in translating recommendations into implementation.

*“I have no authority to find out whether [the head of] district health office implements any recommendation. My responsibility as the coordinator of the MDA team is merely to give a recommendation, and I have played my part.”*

(A management team, 45 years old)

The management team also recognized another challenge due to the implementation of recommendations. It requires an extra budget allocation to provide an action to prevent similar preventable maternal deaths effectively. In this setting, the management team connected poor financial support and the need for extra budget for effective preventive measures. They further exemplified that additional funding was allocated for data collection of maternal deaths occurring in the hospitals outside of patients’ residency area. The perceived lack of financial support should be viewed as poor budgeting, more financial resources are allocated for data collection and the review process than initially intended.

*“I wish we had financial resources [to implement] the recommendation. Another challenge was that when maternal deaths occurred in the [hospital] outside of patients’ residential area, this required extra time and cost.”*

(A management team member, 58 years old)

## Discussion

The maternal audit is one of the regularly prioritized programs to support health system decentralization in Indonesia, which commenced in 2001. Indonesia’s decentralized health system, with its many disparity factors among regions, requires extra effort of local assessment to identify the contributing events of maternal deaths, which can drive local evidence into specific actions [15, 16, 20]. This implies that the failure to present an evidence-based recommendation contributes to the event of similar preventable maternal deaths. This study demonstrates the findings on the challenges of providing evidence-based recommendations associated with obtaining accurate data surrounding maternal deaths, reviewing the care provided against evidence-based criteria, and building a mutual partnership between the key hospital decision-makers and district health officers.

Even though the maternal audit guideline in Indonesia emphasizes the importance of the internalization of the ‘no name, no shame, no blame’ principle and involving the existence of the legal framework of the district MDA committee, there is still a gap between regulation and its implementation due to the failure to create a favorable work environment. Other studies also reported similar challenges in internalizing the audit’s principles in developing countries [4, 26, 27].

Our study revealed this negative environment of blaming culture leading to mistrust of the health service provider and stakeholders to the district MDA committee which is consistent with review on inter-organizational collaborations in healthcare work, highlighting the unfavorable effect of unecessary conflict and unachieved tasks on partnership trust resulting in dissolution [28]. Simarly, Lewis highlighted unfavourable environment promotes fear, demoralization, uncertainty and intransparency resulting in failure to determine the lesson-learned and its respective action [20]. Our findings supports this argument implying the impact of poor working relationships between team management, the reviewers, and the health facilities leading to so-called a “vicious circle” instead of a functioning continuous action cycle of maternal deaths. The findings of this study showed such a vicious circle of the sequence of processes involving ignorance of cases, reluctance among the actors of health facilities to disclose confidential medical information, subjectivity in analyzing cases, and lack of support to implement quality improvement in health facilities. In fact, the MDA action cycle was attributed to merely ‘the punitive actions’ in the lower-level facilities.

The negative environment of this study shows what causes the blaming culture related to poor understanding of the process of maternal audit. The lack of available MDA forms aggravated this situation to provide all the necessary information to set up an evidence-based recommendation. This finding is different from other studies showing that the challenges to obtaining complete data were due to inadequacy rather than a shortage of maternal death forms [29] and the failure of health staff to fill out the forms accurately and completely [26, 30]. These findings highlighted the important issue underpinned conducting the review process used unreliable data. The reviewer does not understand the importance of explicit clinical standards of good practice to evaluate the appropriateness of care lead to providing a non-evidence-based recommendation. This study shows that the absence of clinical consensus allows reviewers’ interest to affect the findings due to their reluctance to attribute maternal death to deficiencies in the care provided by their colleagues, especially their seniors. This reluctance in disclosure has also been discussed in a study of the validation of potentially avoidable perinatal deaths conducted in New Zealand [31]. In this present study, the reviewers were hesitant to identify deficiencies of health personnel of higher facilities who were identified as the reviewer’s colleagues and tended to shift the ‘blame’ to the lower-level facilities. This phenomenon can be best described using a proverb of “sharp downward, blunt upward.” This study reflects the argument of French et al. that one crucial approach to implement evidence into practice is to facilitate health professionals to modify their clinical behavior to comply to the evidence-based guidelines [32]. It implies that the failure to coherence the evidence-practice gap in identifying the possible barriers and enablers to develop a specific intervention to health professional groups whose behavior needs changing in reducing preventable maternal death. This finding supports that of a study revealing the challenge of intervention to health workers in Indonesia involving 11 hospitals conducted by EMAS (Expanding Maternal and Neonatal Survival) and POGI that human resource/health worker were frequent contributing factor of maternal death compared to supply, facility, or infrastructure [13].

Although there were barriers in formulating evidence-based recommendations in implementing MDAs at the district level, the government of Central Java has applied the MDG Acceleration Framework (MAF) in 2013. The UN bodies have recognized this initiative framework as the first “provincial MDG action plan with clear targets, indicators, timeline, and budget requirements.” The concepts elaborated in the MAF, including the principles of evidence-based policy-making, represent the action cycle of the MDA that begins with the analysis of the barriers in the implementation of priority interventions according to the conditions of the study setting. This roadmap accommodates the decentralized health system with measurable objectives to improve access and service quality [21].

In this study, the concept of MAF in the action cycle of maternal death review has not been institutionalized by the DHO at the district level. This is likely due to several challenges faced by the district government leadership. First, lack of knowledge of DHO officials and staff was associated with no training due to *frequent* rotation [26, 29]. Nevertheless, the Ministry of Health has conducted training following the revision of the MDA guideline. Parallel with these findings, several studies also showed a lack of knowledge due to no training but not frequent rotation [26, 29]. The results showed that the new officials and staff had none of the required knowledge and skills for their new job description, replacing the trained officials and staff rotated. The rotation of the management team in DHO is carried out by the head of DHO and the Regent/Mayor of the area. Unlike the management team and reviewers, the community health care teams in public health centers had attained sufficient trainings. However, there was an overlapping policy or mismanagement of human resources at the district level indicated by frequent staff rotation leading to lack of knowledge. It implies by not having received a specific decree of appointment, the new rotation of staff had none of the required knowledge and skills for their new job description.

Second, there is an absence of a specific guideline facilitating the translation of recommendations into implementation. The findings support the review by Pattinson and Bergh in 2008, highlighting that recommending solutions for the policy problems does not necessarily lead to implementation [24]. In the study setting, the national guideline provides general recommendations without specific strategies to ensure the outcome achievement at the local level. Other studies also reported similar findings that there is no specific guideline to design an implementation intervention and how to properly approach it, making it continuously a challenge to overcome [33, 34]. As seen in our study, these deficiencies lead to some inappropriate actions and supervision of the DHO to improve maternal health access and service quality. From the viewpoints of cross-sectoral collaboration, to solve the bottlenecks of the delays of maternal deaths, the DHO required external support to advocate critical policy reforms and overcome identified constraints of maternal health in the region. This finding suggests the importance of trust in maintaining collaborations between functional bodies in voluntary or compulsory programme [28].

Forging ahead, establishing a workable design of maternal death audits to provide a local framework for local problem-solving is an essential step for quality improvement. A decentralized health system in Indonesia, with regional capacity disparities, providing local evidence-based recommendations of MDR can be implemented to be the gold standard for understanding the actual burden of maternal mortality at the district level. In addition, the decentralized system requires extra effort to ensure outcome achievement, such as the implementation of mentoring programs at the district level. Evidence-based policymaking at the district level can be intended to reduce preventable maternal mortality in their region and meet their specific needs.

### Strengths and limitations

The strengths of this study are is that it evaluated the challenges in implementing the national program in a decentralized system to reduce maternal mortality. Therefore, our findings represent the actual experiences of the MDA committees at the district level to identify the local contributing factors of maternal deaths and translate the general national recommendations into specific solutions. Our sample selection specifically included the districts conducting programs to reduce maternal mortality, such as training of basic and comprehensive obstetrics and neonatal care to personnel of public health centers and referral hospitals and mentoring programs to the health facilities from government organizations and different non-government organizations.

One of the limitations is that the MDA committees were recruited primarily from districts in rural areas in Java island. Therefore, the findings may not be transferable to MDA committees in urban settings and Eastern Indonesia. In Central Java, three municipalities with educational centers (universities) provide external reviewers for the maternal death audit process. While in eastern Indonesia, different geographic, demographic, and cultural characteristics can capture other challenges in conducting MDA [35, 36]. However, this study did not delineate the special concerns of these settings, which may or may not face the same challenges to develop evidence-based recommendations through standardized reviews of maternal deaths.

## Conclusions

This study has determined a critical barrier to provide evidence-based recommendations for adaptive practices in reducing maternal mortality at the district level and has provided a foundation for further research in the institutionalization of a workable MDA. To eliminate the blaming culture, we recommend developing a framework and a tool of maternal audit and reviews as a set of evidence-based recommendation that enables guidance towards reliable intervention and support the decentralized health system at the district level. Furthermore, due to the challenges of specific characteristics, geography, demography, and culture, these methods and findings can be applied for further research to identify the barriers of MDA in Eastern Indonesia and remote areas, which can deliberate a basic design to improve the quality of MDA implementation in the area.

## Data Availability

The datasets generated and analyzed during the current study are in Bahasa Indonesia. They are not publicly available due to the confidentiality nature of the participants, but the data can be made available upon request to the authors.

## Abbreviations

MMR: Maternal mortality ratio;
MDA: Maternal death audit;
WHO: World Health Organization;
MDSR: Maternal death surveillance and response;
MDR: Maternal death review;
CEMD: Confidential enquiry of maternal death
SUPAS: Inter-census population survey
MDGs: Millenium development goals;
EMAS: Expanding maternal and neonatal survival;
POGI: Indonesian Society of Obstetrics and Gynecology
EmOC: Emergency obstetrics care;
FGDs: Focus group discussions;
DHO: District health office;
MOU: Memorandum of understanding
